# Dental Fear and Concomitant Factors in 3-6 Year-old Children

**DOI:** 10.5681/joddd.2012.015

**Published:** 2012-06-06

**Authors:** Katayoun Salem, Maryam Kousha, Arash Anissian, Asadollah Shahabi

**Affiliations:** ^1^Assistant Professor, Department of Pediatric Dentistry, Faculty of Dentistry, Guilan University of Medical Sciences, Rasht, Iran; ^2^Assistant Professor, Department of Pediatric Psychiatry, Faculty of Medicine, Guilan University of Medical Sciences, Rasht, Iran; ^3^Associate Professor, Manager of Research, Shahid Beheshti University of Medical Sciences, Tehran, Iran

**Keywords:** Behavior management, child, dental fear and anxiety, general anxiety

## Abstract

**Background and aims:**

Dental fear/anxiety as a barrier in accessing oral health care is poorly investigated in Iranian children. The aims of this study were to evaluate the prevalence of dental fear and behavior management problems, as well as to examine the relationship between dental fear/anxiety and probable concomitant factors.

**Materials and methods:**

Mothers of 200 children aged 3-6 were participated in this descriptive-analytic study, and completed the CFSS-DS, SDQ, Chora and Spielberger questionnaires for both child and parents’ general and dental anxiety in this descriptive- analytic study. Behavior was evaluated according to Frankl scale. Statistical approaches included T-test, chi-sq, and Pearson Linear correlation.

**Results:**

The mean score of dental fear was 32.15 ± 10 and the prevalence was 22.2%. Significant correlations were found between child’s dental fear, general fear and behavior management problems; however, no relationship was found between child’s dental fear and parental dental or general fear.

**Conclusion:**

According to results of this study dental fear/anxiety seems to be more conditional and related to child’s temperament than parental impact. Parental evaluation of dental fear can be used as a predictor of child’s dental behavior.

## Introduction

Avoiding behavior in a dental setting has been attributed to a number of factors including dental fear/anxiety (DF/DA). The terms dental fear and dental anxiety are often used synonymously,^1^ and considered to be the main reason of behavior management problems and avoiding dental care.^2^These problems sometimes urge the dentist to substitute the conventional treatment with more complicated alternatives such as sedation or general anesthesia. It is reported that 5%,^3^ to 52%,^4^ of the children experience such degrees of dental fear/anxiety that makes providing treatment difficult. Although mild fear is a normal expectation during the child’s development, when the extent is disproportionate to natural threat, the problems are evolved. Fearful patients, based on the origin of their fear fall into two broad distinctions: exogenous and endogenous. It is believed that the exogenous type of fear relates to a direct or indirect traumatic experience^1 , 2^ while the latter may be a component of a constitutional vulnerability to anxiety disorders.^5^ Despite of the improvements in pain control and treatment modalities, dental fear and anxiety has remained relatively constant during the last 50 years.^2^

There are several measurement techniques to assess dental fear and behavior management problems including: (a) behavioral ratings by direct observation of the behavior during dental treatment, (b) report of anxiety by child or accompanying parent using psychometric scales, (c) physiological measures, and (d) projective techniques. Dental subscale of Children’s Fear Survey Schedule (CFSS-DS) is an example of psychometric measures that has been used extensively. Parental version is normally used for children under age 13.^8^

Considering sparse data on pediatric dental fear in Iran, the purpose of present study was (1) to investigate the prevalence of dental fear and behavior management problems among 3 to 6 year-old Iranian children, and (2) to assess the concomitant factors such as children’s general anxiety, “mothers’ dental fear and general anxiety status” and their relationship with dental fear and behavior management problems.

## Materials and Methods

Two hundred consecutive 3-6 year old pediatric patients, attending the dental clinic of Guilan University of Medical Sciences, Rasht, Iran, from November to June 2009, participated in this cross-sectional study. The participants had a former restorative appointment in this center at the time of completing the questionnaire. The exclusion criteria were known psychiatric disorders or disabilities. However, no one was excluded. The sample size was calculated based on α=0.05, β=0.2, σ ^2 ^=4, d=0.4, and a number of 197 subjects were determined.

The study was approved by Ethical committee of Guilan Dental School Research Center. After receiving the parental consent and explaining the questionnaire to them, the parents (mostly mother), were asked to complete questionnaires in the waiting room, except for the CFSS-DS, which was completed during the child’s restorative treatment. The responding parent was the mother in the majority of instances, and classified from low to medium low socioeconomic status according to their education and social position.

Data was analyzed by SPSS 14 statistical software (SPSS Inc., Chicago, USA), and Sample t-test, chi-square, Pearson’s linear correlation were used for data analysis.

The assessment of children’s dental fear (DF) was performed through the means of parental version of Child Fear Survey Schedule- Dental Subscale (CFSS-DS) by mothers. It is a likert type 5 pointed scale from 1 (non afraid at all) to 5 (very afraid) and the scores from 15 to 75. The cutoff score for CFSS is determined ≥38 that its reliability and validity in Persian language has been verified previously by Ghasempour et al.^9^

To assess child’s general anxiety, the “Strength and Difficulties ties Questionnaire” (SDQ) developed by Robert Goodman was used. This is a 25 item, one paper questionnaire with three response categories (not true, somewhat true, and certainly true). Each question scores between 0-2 and the final score ranges between 0-50. A Score of >13 indicates child’s general anxiety. The SDQ has been translated into Persian under supervision of Goodman. The SDQ is a brief questionnaire used to screen the psychiatric disorders among children and adolescents. It also detects the probable distress or social impairment of the child, which would be caused by the symptoms. This scale prevents overestimating the detection of childhood psychiatric disorders. The SDQ picks up both positive and negative behaviors.^10^

The next questionnaire was the Persian version of Corah’s Dental Anxiety Scale (DAS) which was used to assess the parental dental anxiety.^11^ It is a reliable, valid and useful measure of anxiety for adults. This questionnaire asks patients to rank their anxiety on a scale of 1 to 5 for each of 4 situational dental questions. Scores of ≥ 13 indicate dental anxiety in adults.^7^

Finally, parental general anxiety was evaluated by Persian version of Spielberger State-trait anxiety scale.^1^ The mothers were asked to report their feelings on the moment of completing questionnaire. The intensity of state anxiety was determined by 4 scores ranged from very low to very high.

The questionnaires were revised before receiving for any probable uncompleted parts and included if they had less than 30% missing values 4 questions and missing items were compensated with item mean values.^1^Behavior management problems were assessed according to Frankl Behavioral Rating Scale by one investigator (AS).

## Results

A total of 200 mothers completed the questionnaires. Demographic status of study population is presented in Table 1. The study group fell into low/medium low socioeconomic status according to parental educational and occupational status. Mean values of CFSS-DS, SDQ, DAS and Spielberger are presented in Table 2. All the values fell in normal limits. The only exception was maternal general anxiety which categorized as moderate anxiety.

**Table 1:** Demographic status of study participants

Item	N (%)
Gender Male Female	106 (53%) 94 (47%)
Age 3-4 4-5 5-6	41 (20.1%) 59 (29.6%) 100 (50.3%)
Paternal educational level High school or less College University	146 (73%) 37 (18.5%) 16 (8%)
Maternal education High school or less College University	168 (84%) 25 (12.5%) 7 (3.5%)

College’s level of education means two years after diploma

**Table 2:** Mean scores of child and mother dental and general anxiety

Item	Mean± sd	Normal Moderate
Child dental fear	32.15±10.9	<38 26
Child general fear	12.11±4.26	<13 8
Mother dental anxiety	9.38±3.4	<13 8
Mother general anxiety	39.7±10.12	20-31 32-42

The mean child’s dental fear score was 32.15 (SD=10.9) and the prevalence was 22%. The highest ranks belonged to injection and drilling for both genders. The cutoff score for child’s dental fear is >38.

Girls showed significantly higher scores 33.92 (SD=12.3) than boys 30.57 (SD=10.1) (t-test, P=0.031, Mean Difference -3.353). However, the correlation between gender and child’s general anxiety was not significant (t-test, P = 0.78).

Table 3 shows the prevalence of dental fear in three age groups (3-4, 4-5, 5-6 yrs). A significant correlation was found between ages 5-6 and dental fear (Pearson Correlation, P = 0.034).

**Table 3:** Prevalence of dental anxiety among three age groups

Age (year)	CFSS-DS
3-4	20%
4-5	22.4%
5-6	23.2%

P=0.034, Pearson correlation CFSS-DS, child fear survey scheduled dental subscale

A positive correlation was found between child’s dental fear and his/her general anxiety (Linear Pearson Correlation test, r=0.47, P<0.001). However, no significant relationship was found between children’s dental fear and maternal dental fear (r=0.186) or general anxiety (r=0.151, Spearman’s correlation).

Evaluation of behavior according Frankl behavioral scale revealed definitely positive behavior in 8.5%, positive 43.5%, negative 36.5% and definitely negative in 11.5% of subjects.

Negative Frankl behavior ratings showed significant relationship with child dental fear (t-test and Levene’s test for equality of variances, P < 0.001), and maternal dental anxiety (t-test, P < 0.001, Mean difference 3.44). However, relationship between negative behavioral and child general anxiety was not significant (t-test, P=0.25).

Parental level of education had no negative impacts on their dental anxiety (chi-square, P=0.50).

## Discussion

In this population of 3-6 year old children from low to medium income families, the prevalence of dental fear/anxiety was similar to a number of previous studies ^14 - 16^ and the CFSS-DS score was similar to but slightly lower than those reported by Wogelius.^17^

DF/A was seen more frequently in girls than boys. This finding is in agreement with those of most studies,^8 , 18 - 20^ although a study has reported opposite results or no differencebetween genders.^21^ However, thisdifference can be a result of biological origin or reflect a response bias as fearfulness is more socially accepted in girls and may be a result of culture and social beliefs.^21^ DF/A was also correlated to child’s general fears,^22 , 17^ which is per se attributed to child’s temperament, and originated from the intensification of childhood fears or can be related to a general anxiety disorders, or blood-injection phobia.^23^ A significant correlation was found between age and DF/A. Interestingly 3-4 years old children showed the lowest prevalence of DF/A. This finding may be attributed to lack of cognitive maturity; because the children have not a clear perception of real fear at this age. Significant reduction in dental fear begins between age 6-7 year and indicates the psychological maturity of child.^3^

Behavior management problems (BMP) as measured by Frankl rating scale, was a common finding in 3-6 age group as observed in 48% of this population. A significant correlation was found between dental fear and uncooperative behavior which was in agreement with previous research,^21 , 24^although another study has reported only a weak correlation.^18^ Both DF/A and BMP are common findings in preschool children. Fear is a natural response when encountering new situations, and behavior problems may be a natural response to fearful situations as well. According to findings of present study, Negative Frankl behavior ratings showed significant relationship with child dental fear. However, these two phenomena have an overlap with each other, as 61% of fearful children have also behavior management problems.^3^Dentists distinguish behavior problems much better than DF/A. Behavior management problems are what the dentist observes, while dental fear is what the patient feels; and the two things do not always correlate. Some children present behavior management problems without having fear, and some children apprehended but are able to cope with situation and finally some children experience dental fear and present behavior management problems. In other words dental fear is only a part of the problems of uncooperative children.^3^

The educational level of parents had no impact on their dental anxiety. In addition the parental level of dental or general anxiety did not seem to influence the child’s dental anxiety.^18 , 19 , 24^ This is in the favor of conditioning theory which attributed the dental fear more to personal experiences rather than parental influence. It is shown that extremely anxious children have a history of treatment in early ages especially for the extraction of the teeth in first appointment and rated the dentist’s behavior as less sympathetic and friendly. ^20 , 25^ It should be remembered that both parental general and dental anxiety in this study population were generally fallen in normal range. With consideration of accompaniment of child’s general anxiety and dental fear, it may be assumed that dentally fearful children may suffer from a more pervasive fear, and may be suggested to refer them for a more comprehensive psychological evaluation, which may improve their general wellbeing as well as dental fear.

## Conclusion

In this population dental fear had a strong correlation with general fear and observed more frequently in girls. The parental ratings of child’s fear was in line with their dental behavior management problems and can be used as an indicator of child behavior.
